# Sequential Immunization with Universal Live Attenuated Influenza Vaccine Candidates Protects Ferrets against a High-Dose Heterologous Virus Challenge

**DOI:** 10.3390/vaccines7030061

**Published:** 2019-07-08

**Authors:** Irina Isakova-Sivak, Victoria Matyushenko, Tatiana Kotomina, Irina Kiseleva, Elena Krutikova, Svetlana Donina, Andrey Rekstin, Natalia Larionova, Daria Mezhenskaya, Konstantin Sivak, Arman Muzhikyan, Anastasia Katelnikova, Larisa Rudenko

**Affiliations:** 1 Department of Virology, Institute of Experimental Medicine, St Petersburg 197376, Russia; 2 Department of Preclinical Trials, Smorodintsev Research Institute of Influenza, St Petersburg 197376, Russia; 3 Department of Toxicology and Microbiology, Institute of Preclinical Research Ltd., St Petersburg 188663, Russia

**Keywords:** universal influenza vaccine, chimeric hemagglutinin, nucleoprotein, live attenuated influenza vaccine, sequential immunization, ferret model

## Abstract

The development of universal influenza vaccines has been a priority for more than 20 years. We conducted a preclinical study in ferrets of two sets of live attenuated influenza vaccines (LAIVs) expressing chimeric hemagglutinin (cHA). These vaccines contained the HA stalk domain from H1N1pdm09 virus but had antigenically unrelated globular head domains from avian influenza viruses H5N1, H8N4 and H9N2. The viral nucleoproteins (NPs) in the two sets of universal LAIV candidates were from different sources: one LAIV set contained NP from A/Leningrad/17 master donor virus (MDV), while in the other set this gene was from wild-type (WT) H1N1pdm09 virus, in order to better match the CD8 T-cell epitopes of currently circulating influenza A viruses. To avoid any difference in protective effect of the various anti-neuraminidase (NA) antibodies, all LAIVs were engineered to contain the NA gene of Len/17 MDV. Naïve ferrets were sequentially immunized with three doses of (i) classical LAIVs containing non-chimeric HA and NP from MDV (LAIVs (NP-MDV)); (ii) cHA-based LAIVs containing NP from MDV (cHA LAIVs (NP-MDV)); and (iii) cHA-based LAIVs containing NP from H1N1pdm09 virus (cHA LAIVs (NP-WT)). All vaccination regimens were safe, producing no significant increase in body temperature or weight loss, in comparison with the placebo group. The two groups of cHA-based vaccines induced a broadly reactive HA stalk-directed antibody, while classical LAIVs did not. A high-dose challenge with H1N1pdm09 virus induced significant pathology in the control, non-immunized ferrets, including high virus titers in respiratory tissues, clinical signs of disease and histopathological changes in nasal turbinates and lung tissues. All three vaccination regimens protected animals from clinical manifestations of disease: immunized ferrets did not lose weight or show clinical symptoms, and their fever was significantly lower than in the control group. Further analysis of virological and pathological data revealed the following hierarchy in the cross-protective efficacy of the vaccines: cHA LAIVs (NP-WT) > cHA LAIVs (NP-MDV) > LAIVs (NP-MDV). This ferret study showed that prototype universal cHA-based LAIVs are highly promising candidates for further clinical development.

## 1. Introduction

Influenza viruses are highly contagious respiratory pathogens that pose a constant threat throughout the world. In addition to annual epidemics, influenza A viruses can potentially cause pandemics when the new virus differs antigenically from previously circulating variants, meaning the human population is immunologically naïve. The global spread of various subtypes of influenza A viruses in birds provides the preconditions for interspecies transmission: the past two decades have seen an increase in the number of cases of human infection with avian influenza viruses H5, H7 and H9 [1,2,3]. One of the most important initiatives to prepare for an influenza pandemic is focused on developing and evaluating appropriate vaccines. Different approaches and platforms have been used to develop vaccines against potentially pandemic influenza viruses and, over the past few years, a large body of data has been accumulated on the safety and immunogenicity of these vaccines. The latest summary of clinical trials of potential pandemic vaccines can be found on the World Health Organization (WHO) website [4].

This pandemic preparedness plan uses a strain-specific approach, in which candidate vaccine viruses are prepared against the pathogens thought to be most likely to cause the next pandemic. However, as was seen in 2009, such predictions are not always accurate, and fundamentally new approaches are required to develop vaccines that can provide protection against both seasonal and newly emerging potentially pandemic strains. Over the past 20 years, research has been carried out in many parts of the world to develop a universal influenza vaccine that induces broadly reactive and long-lasting immune responses. The general principle of this work is to redirect the adaptive immune response from immunodominant hypervariable regions to low-immunogenic, highly conserved regions of viral proteins [5,6,7,8,9]. Influenza virions contain multiple conservative domains that, because of their functional significance, are rather weak immunogens; classical approaches to immunization are not capable of inducing robust immune responses to these sites [10].

One such conservative antigen region is the stalk domain of the viral hemagglutinin (HA) molecule, which contains epitopes that are highly conserved among influenza A viruses belonging to the same phylogenetic group. Group 1 influenza viruses are those with H1, H2, H5, H6, H8, H9, H11, H12, H13, H16, H17, or H18 HA, while group 2 comprises those with H3, H4, H7, H10, H14, and H15 HA. Several strategies have been proposed to increase the immunogenicity of the HA stalk domain; the most promising one involves sequential immunization with vaccines expressing chimeric HA molecules (cHA) that contain an identical stalk domain (for example, from the H1N1 virus), but in which the globular domains vary significantly in antigenicity, i.e., belong to different subtypes of influenza A viruses [11,12]. This vaccination strategy elicited potent antibody responses against either group 1 or group 2 HAs, with limited cross-reactivity between the two HA groups, suggesting that a cHA-based universal influenza vaccine has to include three components: a group 1 HA, a group 2 HA, and an influenza B stalk-based antigen [13,14].

Several recent studies have demonstrated that live attenuated influenza vaccines (LAIVs) expressing chimeric HAs are capable of inducing cross-reactive HA stalk-specific antibodies, either when used as a priming vaccine followed by inactivated influenza vaccine (IIV) [15,16], or when several cHA-based LAIVs are administered sequentially to mice. With sequential immunization with two or three doses of LAIV, mice were better protected against heterologous challenge infection when the vaccines had the same HA stalk domain, compared with vaccines that contained intact HA [17].

Another promising approach for designing broadly protective influenza vaccines is the development of immunogens that induce strong T-cell immunity, which is known to be cross-reactive among antigenically different viruses [18,19,20]. Although T-cell immunity does not prevent influenza infection, there is evidence that T cells can recognize the conserved epitopes of viral proteins, reduce viral load and alleviate symptoms in animals and humans after infection with heterologous influenza viruses [21,22,23,24]. LAIVs are believed to be good inducers of virus-specific cytotoxic CD8+ T-cell immunity [25,26,27,28,29]. Therefore, sequential immunization with cHA-containing LAIVs should not only induce broadly protective stalk-specific antibodies, but also stimulate cross-reactive T-cell immunity.

However, the cytotoxic T-cell (CTL) immunity induced by licensed LAIVs might be suboptimal in protecting against currently circulating influenza viruses, because the nucleoprotein (NP) and M1 protein, known as the most immunodominant targets for CTL immunity, originate from master donor virus (MDV) isolated over 60 years ago [30,31]. Even though these proteins are relatively conserved among different subtypes of influenza A viruses, they are still subject to slow evolutionary changes [32]. Over the past 20 years, a large number of studies have indicated that certain immunodominant T-cell epitopes have disappeared as the influenza viruses have evolved [33,34,35]. The mutations in the epitopes mean that CTLs targeted to the original, non-mutated epitopes will be inefficient in detecting virus-infected cells [36]. This problem can be partially solved by replacing the NP gene in the genome of the LAIV virus by one from a currently circulating influenza virus. Indeed, recently we showed that engineering of LAIV reassortants with 5:3 genome compositions induced CTL responses that were more relevant to current infections [36,37,38].

In the present study, we evaluated universal LAIV candidate viruses that express chimeric HA molecules and the NP gene of either MDV or wild-type virus in ferrets, the most suitable animal model for studying influenza infection [39]. A sequential immunization regimen was used to assess the ability of the vaccines to induce broadly reactive antibody responses and protect against heterologous virus challenge. A high dose of challenge virus was used in order to evaluate the vaccines’ ability to reduce influenza virus-induced pathology.

## 2. Materials and Methods

### 2.1. Viruses and Proteins

**Figure 1:**
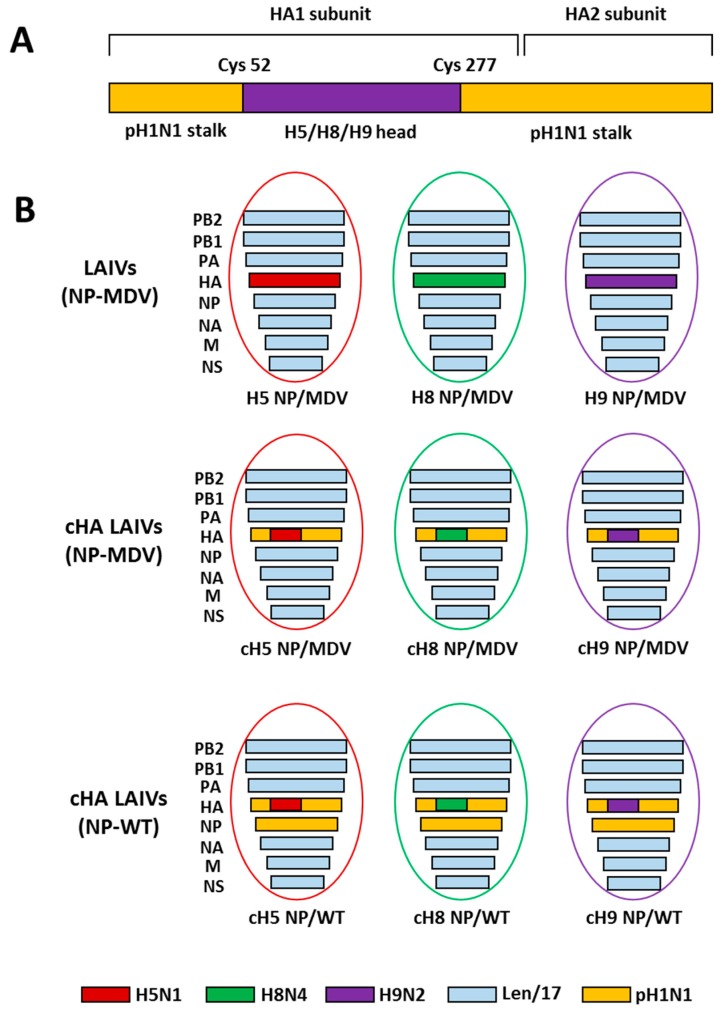
Overview of the LAIV viruses used in this study. (**A**) Schematic representation of the chimeric HA protein structure. (**B**) Genome composition of the LAIV reassortant viruses rescued in this study.

The LAIV reassortant viruses used in this study are shown in Figure 1. All vaccine viruses were generated by standard plasmid-based reverse genetics (RG) on the basis of cold-adapted MDV A/Leningrad/134/17/57 (H2N2) (Len/17) [40]. RG plasmids bearing chimeric HA genes were generated as described previously [17]. The cH5/1 and cH8/1 plasmids contain the stalk domain of A/California/7/2009 (Cal09, pH1N1) virus and the head domains of A/Vietnam/1203/2004 (ΔH5N1) (polybasic cleavage site deleted) and A/mallard/Sweden/24/02 (H8N4), respectively. The cH9/1 plasmid contains the stalk domain of A/South Africa/3626/2013 (SA13, pH1N1) virus and the head domain of A/quail/Hong Kong/G1/1997 (H9N2) strain; the Cal09 and SA13 pH1N1 viruses have 99% amino acid stalk domain identity. RG plasmids bearing control non-chimeric full-length HAs of the H5N1, H8N4 and H9N2 viruses were also generated, as in the previous study [17].

The three groups of LAIV viruses were rescued by electroporation of Vero cells using the Neon transfection system (Invitrogen, USA): (i) classical LAIVs containing non-chimeric HA and NP from MDV; (ii) cHA-based LAIVs with NP from MDV; and (iii) cHA-based LAIVs with NP from pH1N1 virus (Figure 1). Importantly, to avoid any effect of anti-NA antibody on the protection, all nine LAIV strains had the same NA gene from Len/17 H2N2 virus. An additional LAIV virus cH11/1N1 bearing chimeric HA gene (the HA stalk domain of Cal09 and the HA head domain of A/northern shoveler/Netherlands/18/99 (H11N9)) and the NA gene of Cal09 was kindly provided by Professor F. Krammer (Mount Sinai School of Medicine, New York, USA). All LAIV viruses were propagated in 10-day-old embryonated chicken eggs for 3 days at 33 °C. Virus stocks were harvested, clarified by low-speed centrifugation and stored in aliquots at −70 °C.

Wild-type human influenza virus A/South Africa/3626/2013 (pH1N1) was obtained from the influenza virus repository of the National Institute for Biological Standards and Control (NIBSC, London, UK). Avian influenza virus A/herring gull/Sarma/51/2006 (H6N1) was obtained from the influenza virus repository of the Smorodintsev Research Institute of Influenza (Saint Petersburg, Russia). The wild-type viruses were propagated in eggs for 2 days at 37 °C and stored in aliquots at −70 °C.

Recombinant full-length HA proteins, including chimeric cH6/1 (containing HA head domain of A/mallard/Sweden/81/02 (H6N1) and HA stalk domain of Cal09), H3 (from A/Perth/16/2009), H6 (from A/mallard/Sweden/81/02) and H9 (from A/chicken/Hong Kong/G9/1997), were kindly provided by Professor F. Krammer (Mount Sinai School of Medicine, New York, NY, USA). LAH1 recombinant protein representing the long alpha helix of HA stalk domain of Cal09 virus (HA2 residues 52 to 132) was kindly provided by Dr A. Kazaks (Latvian Biomedical Research and Study Centre, Riga, Latvia) [41].

### 2.2. In Vitro Studies

Temperature-sensitive and cold-adapted (*ts/ca*) phenotypes of the LAIV viruses were determined by titration at different temperatures in eggs: 38 °C compared with 33 °C for the *ts* phenotype and 26 °C compared with 33 °C for the *ca* phenotype. Eggs inoculated with 10-fold virus dilutions were incubated for either 72 h (for 33 °C and 38 °C) or 6 days (for 26 °C). In addition, LAIV virus growth was analyzed in Madin-Darby canine kidney (MDCK) cells to determine the 50% tissue culture infectious dose (TCID_50_) on day 4 after inoculation. Virus titers in eggs and MDCK cells were calculated using the Reed and Muench method and expressed in terms of log_10_ 50% egg infectious dose (EID_50_)/mL and log_10_TCID_50_/mL, respectively. A virus was considered to be temperature-sensitive (*ts* phenotype) if the infectious titers at 33 °C were at least 5.0 log_10_EID_50_ greater than at 38 °C. A virus was considered to be cold-adapted (*ca* phenotype) if the infectious titers at 26 °C were not more than 3.0 log_10_EID_50_ lower than at 33 °C [42].

### 2.3. Animals

Male ferrets (*Mustela putorius furo*), aged 5–6 months and weighing 1.1–1.9 kg at the beginning of the experiment, were supplied by Scientific-Production Organization House of Pharmacy JSC (St Petersburg, Russia). They were prescreened by routine hemagglutination inhibition (HAI) test to ensure that they were negative to circulating human influenza viruses and the viruses being tested. Prior to infection, ferrets were randomly selected and housed individually in isolation units with free access to food and water. All animal experiments were conducted using protocols approved by the Local Bioethical Committee of the Institute of Preclinical Research Ltd. (St Petersburg, Russia) (Protocol #BEC 2.12/18 authorized on 28 February 2018). All inoculations, nasal washes and blood sample collections were performed with the animal under short-term anesthesia induced by intramuscular injection of Zoletil 100, 12.5 mg/kg of body weight; every effort was made to minimize suffering. At the end of the study, animals were euthanized with an overdose of Zoletil-xylazine combination.

### 2.4. Ferret Immunization and Challenge

**Figure 2:**
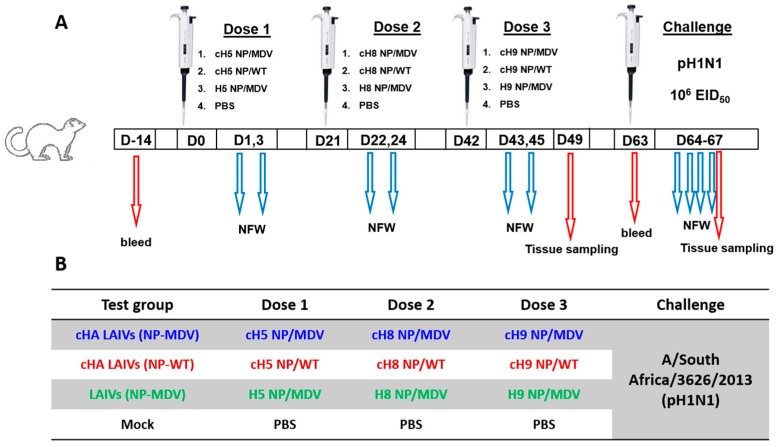
Overview of study design (**A**) and designation of the study groups (**B**).

Four groups of seven ferrets were inoculated intranasally with three doses of LAIV viruses or phosphate-buffered saline (PBS) placebo at 21-day intervals, as illustrated in Figure 2. The vaccine viruses were administered in a dose of 7.0 log_10_EID_50_ and an inoculum of 0.5 mL.

Nasal fluid wash (NFW) specimens were collected on days 1 and 3 after each vaccine dose. Blood samples for serum preparation were collected 14 days before vaccination and on day 63 to assess immunogenicity. Seven days after the last vaccine dose (day 49), three ferrets from each group were euthanized and tissues were collected for evaluation of the safety of the vaccines tested. The remaining four animals from each group were challenged intranasally with 6.0 log_10_EID_50_ of A/South Africa/3626/2013 (pH1N1) virus on day 63, after which NFW samples were collected every day until day 67. On that day, all animals were sacrificed and various tissues were collected to assess viral replication and pathological changes as observed on gross pathology and histopathology.

### 2.5. Virological Methods

Virus replication in the ferrets’ respiratory tract was assessed by endpoint titration of NFW, lung and trachea specimens in embryonated chicken eggs. Tenfold sample dilutions were inoculated into 10–11-day-old eggs and incubated at 33 °C for 72 h (for LAIV viruses) and at 37 °C for 48 h (for the SA13 pH1N1 challenge virus). Virus titers were expressed as log_10_EID_50_/mL for NFW and log_10_EID_50_/gram for trachea and lung specimens. In addition, the pH1N1 viral load in NFW specimens was expressed as the area under the curve (AUC) of the NFW virus titer during the four days post challenge. The AUC was calculated using the trapezoidal rule.

### 2.6. Clinical Signs and Morbidity Outcomes

Ferrets were observed daily for clinical signs (body weight, level of activity, nasal discharge, and sneezing). Nasal symptoms were scored as follows: 1—nasal rattling could be heard or the ferret sneezed during transport from its cage to the evaluation area; 2—there was evidence of nasal discharge on the external nares; 3—the animal exhibited mouth breathing; 0—the animal exhibited none of these symptoms. Activity level was scored over a range from zero to three according to the extent that the animal could be induced to play: 0—the animal was fully playful; 1—the animal responded to play overtures but did not initiate any play activity; 2—the animal was alert but not at all playful; 3—the animal was neither playful nor alert. Scores were summed for each ferret and group medians calculated.

Body temperature was measured using temperature data loggers (Star-Oddi, Reykjavik, Iceland) implanted into the peritoneal cavity and programmed to record body temperature every 30 min. The effect of vaccination or virus infection on the body temperature was expressed as the AUC of the body temperature increase after immunization or challenge (AUC delta T) and as the maximum temperature increase (max delta T). The body temperature increase was calculated by subtracting the baseline temperature (average of the temperatures recorded in periods between vaccinations) from the temperature recorded at any time during the vaccination or challenge phase, excluding the periods of sedations, where a sharp temperature decrease was registered. The AUC was calculated using the trapezoidal rule and the same baseline temperature as mentioned above.

### 2.7. Assessment of Immune Responses

Antibody immune responses were assessed by determining serum antibody titers in an HAI assay and microneutralization test (MNT). In addition, total IgG antibody was quantified by enzyme-linked immunosorbent assay (ELISA). For the HAI assay, serum samples were pretreated with receptor-destroying enzyme (RDE) (Denka Seiken, Tokyo, Japan) to remove non-specific inhibitors and quantified against four HA units of the following viruses: (i) A/South Africa/3626/2013 (pH1N1); (ii) H5 NP/MDV LAIV; (iii) H8 NP/MDV LAIV; (iv) H9 NP/MDV LAIV; (v) cH11/1N1 LAIV; (vi) A/herring gull/Sarma/51/2006 (H6N1). The HAI assay was performed as described elsewhere [43] using a 0.5% suspension of chicken red blood cells (RBCs). Additional HAI assays were performed with H5 NP/MDV LAIV and H8 NP/MDV viruses using 1.0% suspension of horse RBCs, because very low HAI antibody titers were detected when chicken RBCs were used. The samples with a negative HAI result at a 1:10 dilution were assigned the value 1:5 for the purpose of statistical analysis.

MNT was performed with homologous viruses H5 NP/MDV LAIV, H8 NP/MDV LAIV and H9 NP/MDV LAIV, as well as with heterologous strains cH11/1N1 LAIV and SA13 (pH1N1), as described by He et al. [44] with minor modifications. Briefly, two identical 96-well plates containing ferret sera were prepared with twofold serial dilutions of RDE-treated serum samples in growth medium (DMEM (Gibco) supplemented with 1× antibiotic–antimycotic (Gibco) and 1 μg/mL TPCK trypsin (Sigma, St. Louis, MO, USA), in a volume of 50 μL. An equal volume of virus at a concentration of 100 TCID_50_ was added to one of the plates. Following incubation for one hour at 33 °C, the virus–serum mixtures were transferred to a 96-well plate containing a confluent monolayer of MDCK cells and incubated for an additional 2 h at 33 °C in 5% CO_2_. The plates were then washed three times with PBS and the serum dilutions from the second 96-well plate were transferred to the MDCK cells. After overnight incubation at 33 °C in 5% CO_2_, the medium was removed, the cells were washed with PBS and were fixed with cold 80% acetone for 20 min. Cell-based ELISA was then used to quantify the expression of influenza A virus nucleoprotein. The fixed cells were first blocked with PBS containing 5% non-fat milk (PBS-M) for 30 min at room temperature and quenched with 3% hydrogen peroxide for 20 min at room temperature. After three washes with PBS/0.01% Tween-20, 50 μL of horseradish peroxidase-conjugated monoclonal antibody to influenza virus NP, diluted 1:4000 in PBS-M (Enterprise for the Production of Diagnostic Products Ltd., Saint Petersburg, Russia), was added to each well. Antibody binding was detected with 3,3′,5,5′-tetramethylbenzidine (TMB) Substrate Reagent Set (BD Biosciences, San Jose, CA, USA). The 50% MNT (MNT_50_) titer was defined as the last serum dilution with optical density (OD) values lower than a cut-off value (CV) determined by the following equation:

$$\mathrm{CV}=\frac{\left(\mathrm{mean}\;\mathrm{OD}\;\mathrm{of}\;\mathrm{virus}\;\mathrm{only}\;\mathrm{wells}\right)-\left(\mathrm{mean}\;\mathrm{OD}\;\mathrm{of}\;\mathrm{uninfected}\;\mathrm{wells}\right)\;}{2}+\left(\mathrm{mean}\;\mathrm{OD}\;\mathrm{of}\;\mathrm{uninfected}\;\mathrm{wells}\right)$$

The samples with an OD value higher than the CV in the first well with a 1:10 serum dilution were assigned the value 1:5.

ELISA was performed as described elsewhere [45], with some modifications. Briefly, high-sorbent 96-well plates (Greiner, Germany) were coated with 100 µL of either 16 HA units of sucrose-purified whole virus or 2 µg/mL of a recombinant protein in carbonate-bicarbonate buffer, in a volume of 100 µL per well overnight. The following proteins were used as antigens in the ELISA: full-length H3, H6, H8 and H9 HA proteins: chimeric cH6/1 protein: and LAH1 protein. Twofold dilutions of sera were prepared starting from 1:10 and added to the coated wells, which were then incubated with anti-ferret IgG conjugates to horseradish peroxidase supplied by Sigma (St. Louis, MO, USA). Antibody binding was detected with TMB Substrate (BD Biosciences, San Jose, CA, USA). The antibody titers were defined as the last dilution with OD_450_ values more than 3 SD greater than the mean OD_450_ measured in control wells (all components except for the serum specimens). The samples with an OD value lower than the mean OD+3SD in the first well with 1:10 serum dilution were assigned the value 1:5. A fourfold or higher increase in antibody titer after vaccination was regarded as a positive antibody response.

### 2.8. Pathology Studies

At the time of necropsy, a complete macroscopic (gross pathology) examination was performed. The nasal turbinates (NT), trachea and lungs were studied in detail, and the abdominal and pelvic cavities were examined. Tissue sections of nasal turbinates, trachea and left cranial and left caudal lung lobes were taken from each sacrificed animal on day 4 post-challenge and used for histological analysis. After fixation in 10% buffered formalin, lungs were embedded in paraffin and prepared for histopathological analysis. For nasal turbinates and trachea, after fixation in formalin the specimens were decalcified in Gooding-Stewart fluid (a mixture of 25% concentrated formic acid, 5% formalin solution and 75% distilled water) for 14 days and then embedded in paraffin and prepared for histopathological analysis. Tissue sections of 5 µm were prepared and stained with Alcian blue at pH 2.5, followed by hematoxylin and eosin staining to reveal goblet cells on microscopic examination. For the assessment of fibrin deposition, sections of lungs were stained with picro-Mallory stain [46]. Infection pathology was evaluated as described elsewhere [47], including indicators of damage to the epithelial lining and acute inflammation. The damage to lung tissues was scored using epithelial hypertrophy, epithelial hyperplasia, pseudosquamous epithelium, and epithelial necrosis of bronchi and bronchioles, as well as hypertrophic pneumocyte type 2, hyperemia septa, alveolar emphysema, and alveolar hemorrhages. Acute inflammation in lungs was assessed in terms of bronchitis, bronchiolitis, peribronchitis, peribronchiolitis, interstitial infiltrate, alveolitis, vasculitis, and perivasculitis. The infection damage to nasal turbinates was assessed as epithelial damage, hyperemia, lymphocytic infiltrate, exudate, and edema. Each of these parameters was scored as: 0 absent; 1 minimal; 2 slight; 3 moderate; 4 strong; and 5 severe. The sum of the parameter scores for damage and inflammation was used for statistical analyses to estimate the degree of vaccine-induced protection. At least six microscopic fields were scored for each lung specimen. The percentage of affected tissue was estimated from the sections as a measure of the extent of damage.

### 2.9. Statistical Analyses

Data were analyzed with Statistica software (version 6.0; Statsoft Inc., Tulsa, OK, USA). The statistical significance of the differences in pathology estimates, as well as in viral titers in eggs, MDCK cells, NFW specimens and tissues of ferrets was assessed using the Mann-Whitney U-test. Differences in log_2_-transformed HAI, MNT_50_ and ELISA antibody titers were also subjected to the Mann-Whitney U-test. *p* values of <0.05 were considered significant. Correlations between antibody immune responses and pathology outcomes after challenge were assessed by nonparametric analysis (Spearman rank test) using GraphPad Prism version 6.01 (GraphPad Software, Inc., San Diego, CA, USA).

## 3. Results

### 3.1. Virological Characterization of LAIV Viruses Used in the Study

The phenotypic properties of the recombinant LAIV viruses containing cHAs and/or wild-type nucleoprotein generated for this study were compared with those of the analogous LAIVs containing classical full-length HA. LAIV viruses are characterized by *ts/ca* phenotypes, i.e., they actively replicate at low temperature (26 °C), but their replication at high temperature (38 °C) is impaired. The replacement of the HA in classical LAIVs with cHA, as well as the replacement of Len/17 NP with that of pH1N1 virus, did not affect the *ts* phenotype: all cHA LAIVs replicated poorly at high temperature in eggs (Table 1). Five of six cHA LAIVs also preserved the *ca* phenotype; the cH5 NP/WT candidate that contained both chimeric HA and wild-type NP replicated poorly at 26 °C; the difference in titer compared with growth at 33 °C was 4.5 log_10_EID_50_. Interestingly, the analogous cH5 containing the NP from MDV preserved its *ca* phenotype (the difference in viral titer between 33 °C and 26 °C did not exceed 3.0 log_10_EID_50_). These data suggest that the source of the NP gene in the LAIV virus could affect the *ca* phenotype. However, the effect is strain-specific, as the other stalk-based LAIVs—cH8 NP/WT and cH9 NP/WT—replicated efficiently at low temperature in eggs (Table 1).

Importantly, all engineered LAIV candidates replicated efficiently in eggs in optimal conditions, though for one virus, cH5 NP/WT, the endpoint titer was significantly lower than that of the classical counterpart. Since the analogous cHA-containing LAIV with MDV NP had a titer similar to that of the control virus, it is likely that the wild-type NP was responsible for the lower viral titers in eggs. The replication profile of the LAIV viruses in MDCK cells was affected by the origin of both the HA stalk domain and the NP gene. Both LAIV candidates that had the cH5 HA molecule showed significantly lower virus titers than their control LAIV expressing natural HA (Table 1). These results indicate that the inclusion of an irrelevant stalk domain in H5 molecules can reduce the ability to replicate in mammalian cells, without affecting growth activities in eggs. The effect of the pH1N1 stalk domain was strain-specific, since cH8 NP/MDV and cH9 NP/MDV LAIVs had similar titers in MDCK cells as the H8N2 and H9N2 LAIVs, respectively. An effect of wild-type NP on virus titer in MDCK cells was noted for all three pairs of LAIV viruses. However, the effect varies: for the cH5- and cH9-containing LAIVs, the WT NP decreased virus titer (7.3 vs. 6.4 log_10_TCID_50_/mL, *p* = 0.049, and 8.2 vs. 5.2 log_10_TCID_50_/mL, *p* = 0.044, respectively); for the cH8-containing LAIVs, the WT NP increased viral replication in mammalian cells (5.8 vs. 7.0 log_10_TCID_50_/mL, *p* = 0.029).

### 3.2. Safety Testing of Universal LAIV Candidates in Ferrets

**Figure 3:**
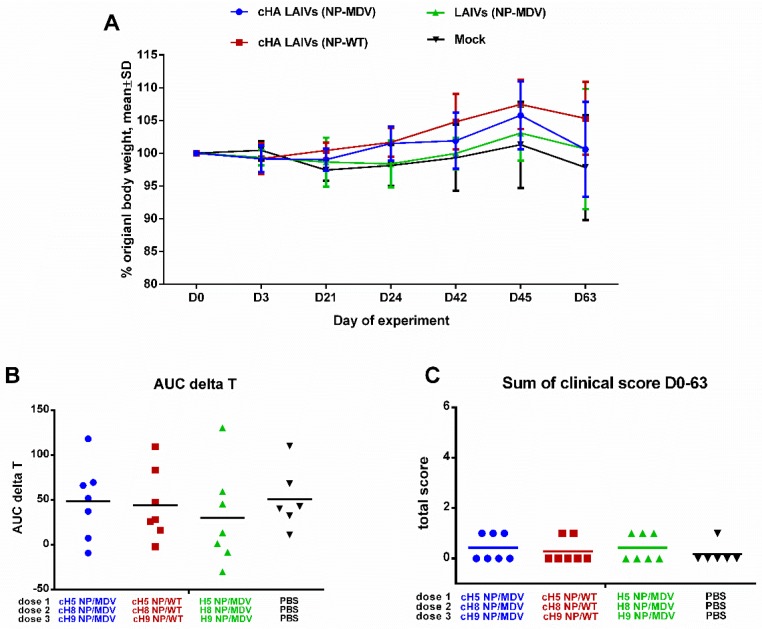
Safety of sequential immunization of ferrets. (**A**) Percentage body weight change. (**B**) Area under the curve of delta T relative to baseline temperature for each ferret over the immunization phase. (**C**) Scores for clinical signs of influenza infection in ferrets throughout the immunization period. No significant differences were observed for any of the parameters.

Safety evaluation in a ferret model is one of the essential steps in preclinical trials of any new influenza vaccine [39]. There were no significant differences in clinical scores or percentage body weight loss at any time, and the overall body temperature fluctuations over the immunization phase were similar in the three LAIV study groups (Figure 3). The vaccines were therefore considered to be safe and well tolerated. These data suggest that neither chimeric HA nor wild-type NP influenced the attenuated phenotype of LAIV viruses; this was in concordance with preservation of temperature-sensitive phenotype, which is the main correlate of attenuation for cold-adapted LAIV viruses.

### 3.3. Shedding and Immunogenicity of Universal LAIV Candidates in Ferrets

**Figure 4:**
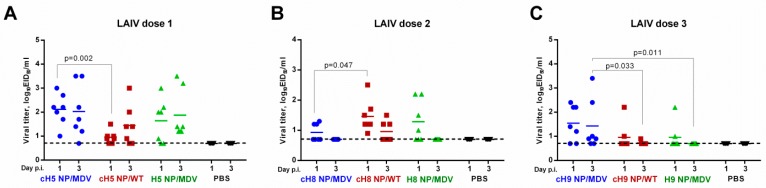
Replication of LAIV viruses in the upper respiratory tract of immunized ferrets after the first (**A**), second (**B**) and third (**C**) vaccine dose.

All nine LAIV reassortant viruses replicated in the upper respiratory tract (URT) of intranasally immunized ferrets, to different extents. The first immunization with H5-based LAIVs yielded similar NFW titers on day 3 post infection. However, the cH5 NP/WT vaccine replicated more slowly than the cH5 NP/MDV vaccine: the day 1 NFW titers in the two LAIV groups were significantly different (Figure 4A). These data suggest that the NP source can affect the kinetics of LAIV virus growth in mammalian cells, which is in concordance with the differences in cH5 NP/WT and cH5 NP/MDV titers in MDCK cells (Table 1). An opposite effect was noted for the cH8-based LAIVs: the vaccine with wild-type NP replicated more efficiently in the upper respiratory tract of ferrets on day 1, than the cH8 NP/MDV virus (Figure 4B). These data also correlated with the efficiency with which the two LAIV strains replicated in MDCK cells (Table 1). In general, all three H8-based LAIVs given at the second vaccination replicated to lower titers than the H5-based LAIVs given at first vaccination and were barely detected by day 3 after inoculation. Of the three H9-based LAIVs given as the third dose, the cH9 NP/MDV was the most infectious and replication was more prolonged than that of the other two LAIVs (Figure 4C). Again, this better replication efficiency in the ferret URT correlated well with virus infectious titers in MDCK cells, suggesting that *in vitro* viral titer in a mammalian cell line can be a good predictor of LAIV virus replication in the respiratory tract of animals. Overall, it can be concluded that the immunity induced to the previous vaccination(s) was not sterile; LAIV viruses with heterologous HA head domains were able to overcome this immunity and replicate after the second and third immunizations. As expected, no infectious viruses were detected in the placebo group (Figure 4).

**Figure 5:**
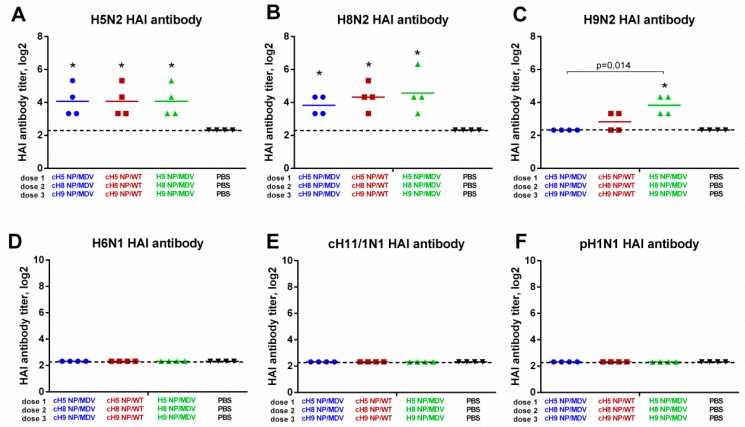
HAI antibody immune responses in immunized ferrets measured against a panel of viruses. (**A**) H5 NP/MDV LAIV. (**B**) H8 NP/MDV LAIV. (**C**) H9 NP/MDV LAIV. (**D**) A/herring gull/Sarma/51/2006 (H6N1) wild-type virus. (**E**) cH11/1N1 LAIV virus bearing chimeric HA gene (the HA stalk domain of Cal09 and the HA head domain of A/northern shoveler/Netherlands/18/99 (H11N9)) and the NA gene of Cal09. (**F**) A/South Africa/3626/2013 (pH1N1) wild-type virus. The HAI titers for H5 and H8 antigens were determined using horse red blood cells; the remaining antigens were tested with chicken RBCs. * indicates significant difference with the mock-vaccinated group. The dashed line indicates the limit of detection.

On day 63, HAI antibody titers were measured against a panel of influenza A viruses (Figure 5). Interestingly, with chicken red blood cells HAI antibody titers against H5N2 and H8N2 were almost undetectable, whereas using horse red blood cells in the assay revealed significantly increased HAI titers in all three LAIV test groups, indicating that the immunizations were successful (Figure 5A,B). The H9N2 LAIV virus did not agglutinate horse RBCs, and was tested with chicken RBCs. The only significant increase in anti-H9N2 HAI antibody titer was seen for the LAIV (NP-MDV) group, although the H9 NP/MDV virus given as the third LAIV dose in this group replicated poorly in the upper respiratory tract of immunized ferrets (Figure 4C). As expected, no HAI antibody against heterologous influenza viruses (such as H6N1, cH11/1N1 and pH1N1) was found in the ferret sera on day 63 (Figure 5D–F).

**Figure 6:**
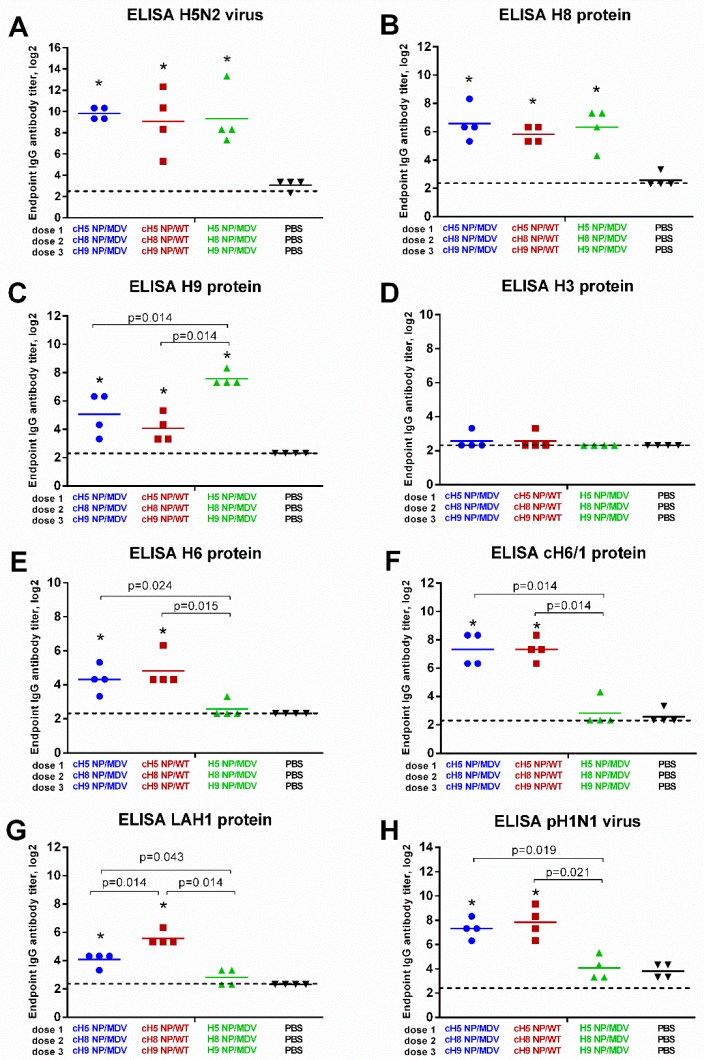
Cross-reactivity of the induced antibody responses. Serum IgG endpoint antibody titers in immunized ferrets were measured by ELISA against: H5 NP/MDV LAIV whole virus antigen (**A**); H8 full-length HA protein (**B**); H9 full-length HA protein (**C**); H3 full-length HA protein (**D**); H6 full-length HA protein (**E**); pH1N1 whole virus antigen (**F**); cH6/1 chimeric HA protein (**G**); and long alpha helix 1 (LAH1) recombinant protein (**H**). * indicates significant difference with the mock-vaccinated group. The dashed line indicates the limit of detection.

Serum IgG antibodies detected by ELISA on day 63 of the experiment were much higher than the HAI titers. When H5 NP/MDV LAIV whole virus was used as antigen, the endpoint IgG antibody titers exceeded 1:1024 in all three vaccine groups, confirming that the immunizations were successful (Figure 6A). Significant rises in IgG titers were also seen for the recombinant H8 and H9 protein antigens, which also indicates successful immunization, as all the vaccine groups included reassortant viruses containing the HA head domains from H8N4 and H9N2 viruses (Figure 6B,C). Consistent with the HAI assay results, the LAIV (NP-MDV) vaccine group induced higher IgG antibody against H9 antibody than the two LAIV groups expressing chimeric HAs (see Figure 5C and Figure 6C), suggesting that the H9-specific IgG antibody mostly binds the HA head domain near the receptor binding site, thus interfering with RBC agglutination. As expected, no IgG antibody was detected against H3 HA protein, since this antigen belongs to the group 2 HA influenza viruses, which have little cross-reactivity with the viruses in group 1. Interestingly, vaccination with cHA-based LAIVs induced significant levels of H6-reactive antibodies that were not active in HAI assay (Figure 5D and Figure 6E), suggesting that these antibodies bind to other parts of the virus HA than the head domain. In most previous studies, HA stalk-specific IgG antibody titers have been measured in ELISA using chimeric HA protein containing the stalk domain of interest (i.e., from pH1N1 virus) and an irrelevant globular head domain (i.e., from H6N1 virus) [48]. Here, we measured stalk-specific antibody levels in ELISA with recombinantly produced chimeric cH6/1 protein. Only the cHA-expressing LAIVs were able to elicit significant levels of stalk-reactive antibody; classical LAIVs did not (Figure 6F). We also measured the binding of serum IgG antibody with LAH1 protein, which is the recombinant protein representing the long alpha helix of the HA molecule of pH1N1 influenza virus. As for the cH6/1 protein, significant increases in LAH1-binding antibody levels were observed for the two cHA-based LAIV sets, but not for classical LAIVs (Figure 6G). Surprisingly, the LAH1-binding endpoint IgG antibody titers were higher in the ferrets vaccinated with cHA LAIVs (NP-WT) than in those given cHA LAIVs (NP-MDV), suggesting that the population of HA stalk-binding IgG antibody can be diverse and bind to different parts of the HA stalk domain. Finally, the endpoint IgG antibody titers against whole pH1N1 virus were determined in ferret sera. Since the HA molecule of this virus contains a stalk domain nearly identical to the cHA-based LAIVs and the HA head domain was not present in the LAIV viruses, the HA-binding antibody most probably binds to the HA stalk domain, rather than the head domain. Indeed, pH1N1-binding IgG antibody was significantly induced only by the cHA-expressing LAIVs, and not by classical LAIVs (Figure 6H). Overall, the ELISA results clearly indicate that the source of stalk domain in LAIV viruses significantly affects the ability to induce stalk HA-reactive antibody after sequential immunization.

**Figure 7:**
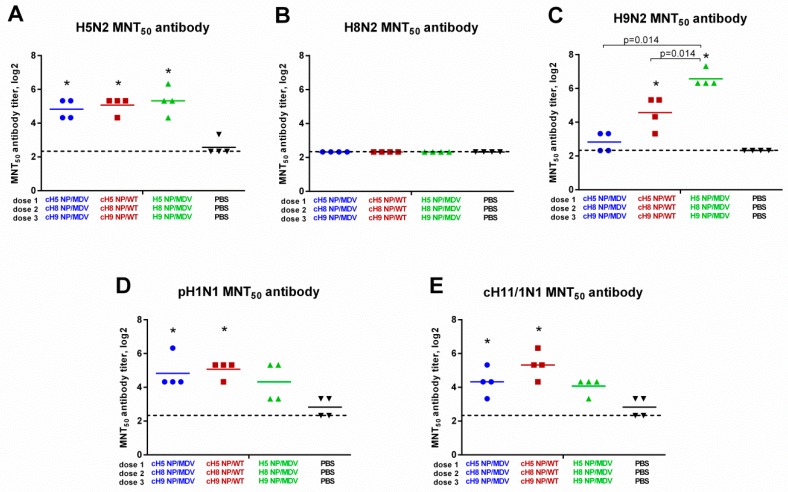
Neutralizing activity of the induced antibody responses. Broadly neutralizing antibody responses in immunized ferrets were measured in 50% microneutralization test (MNT_50_) against: H5 NP/MDV LAIV virus (**A**); H8 NP/MDV LAIV (**B**); H9 NP/MDV LAIV (**C**); A/South Africa/3626/2013 (pH1N1) virus (**D**); and cH11/1N1 LAIV virus (**E**). * indicates significant difference with the mock-vaccinated group. The dashed line indicates the limit of detection.

It is known that ELISA only measures antigen binding and cannot predict whether the antibodies are functional. To assess the functional activity of the induced antibody immune responses, sera from immunized ferrets was tested in a microneutralization test using the three homologous viruses (H5N2, H8N2 and H9N2), as well as two heterologous influenza viruses: cH11/1N1 LAIV and the SA13 pH1N1 wild-type virus. Replication of H5N2 virus in vitro was similarly inhibited by sera from all three LAIV vaccination groups, in line with the results of HAI and ELISA assays (Figure 7A). Furthermore, inhibition of H9N2 virus was also correlated with the results of HAI and ELISA: the MNT_50_ titers were significantly higher in the classical LAIV group, compared with the two cHA-expressing LAIV groups (Figure 7C). Surprisingly, the H8-specific antibody revealed in the HAI assay (Figure 5B) and ELISA (Figure 6B) were unable to neutralize H8N2 LAIV replication in vitro (Figure 7B). This is probably because the intrinsic properties of the H8 subtype influenza virus require higher antibody titers for neutralization, and one dose of LAIV is not sufficient to induce such a high antibody response. Importantly, the heterologous cH11/1N1 and pH1N1 viruses could be neutralized by the sera from cHA LAIV-immunized ferrets: although the MNT_50_ titers were relatively low (most ferrets did not have a titer above 1:40), both cHA-based LAIV groups showed a significant increase in titer, compared with the control group, whereas MNT_50_ titers in ferrets given classical LAIV were not statistically significantly different from the control group (Figure 7D,E). These data suggest that the cHA-based LAIVs induced broadly neutralizing stalk-specific antibody after sequential immunization, since both the cH11/1N1 and pH1N1 viruses had the same stalk domain as these LAIVs, but differed significantly in the source of HA head domains. It is unlikely that the HA head-targeted antibody can have any cross-reactivity with the H1, H5, H8, H9, and H11 subtypes.

Overall, analysis of serum antibody immune responses in immunized ferrets suggests that the two sets of LAIVs expressing chimeric HA molecules induce a broadly reactive HA stalk-specific antibody, which also possesses neutralizing activity in vitro.

### 3.4. Protective Efficacy of Universal LAIV Candidates against a High-Dose pH1N1 Viral Challenge

#### 3.4.1. Challenge Virus Shedding

**Figure 8:**
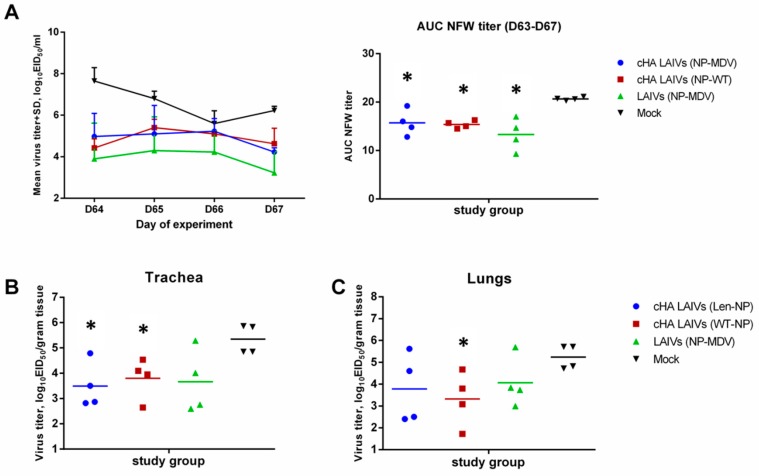
Replication of H1N1pdm09 challenge virus in respiratory tract of immunized ferrets. (**A**) Virus titers in nasal wash fluids at each time point during the challenge phase and the AUC of NFW viral titers for individual ferrets. (**B**) Viral titers in trachea. (**C**) Viral titers in lungs. * indicates significant difference with mock-vaccinated group (Mann-Whitney U test).

Challenge virus was detected on day 4 after infection in all respiratory tissues tested: lungs, NT, and trachea. However, all vaccinated animals shed challenge virus in the URT at significantly lower levels than the mock-vaccinated group (Figure 8A). There was no significant difference between the different vaccination regimens in virus shedding in the URT. However, virus titers in the trachea were significantly lower in animals from the two LAIV groups expressing cHAs, but not for classical LAIVs, compared with the mock group (Figure 8B). Strikingly, a significantly lower pulmonary virus titer was observed only for the animals immunized with cHA LAIVs that contained WT NP (Figure 8C). These data indicate that the replacement of MDV NP with that of WT virus can be beneficial in terms of protection against recent wild-type influenza virus.

#### 3.4.2. Body Weight Loss and Clinical Manifestations of Disease

**Figure 9:**
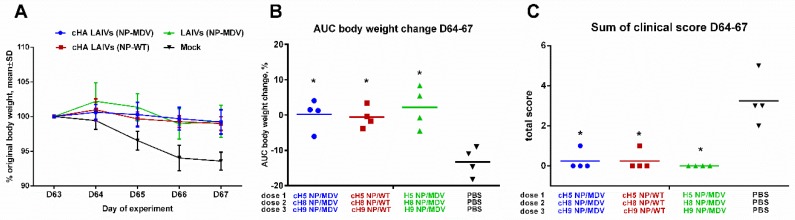
Clinical signs of influenza infection in immunized ferrets after challenge. (**A**) Dynamics of body weight change during the challenge phase. (**B**) Area under the curve of percentage body weight change for each ferret. (**C**) Sum of scores for clinical signs for each ferret during the four days after the challenge. * Significant difference from the mock-vaccinated group (Mann-Whitney U test).

All vaccinated animals were protected from body weight loss and clinical signs of disease, as compared with the mock-immunized ferrets (Figure 9). Only the control animals displayed clinical symptoms characteristic of influenza infection, such as sneezing and nasal discharge.

#### 3.4.3. Body Temperature

**Figure 10:**
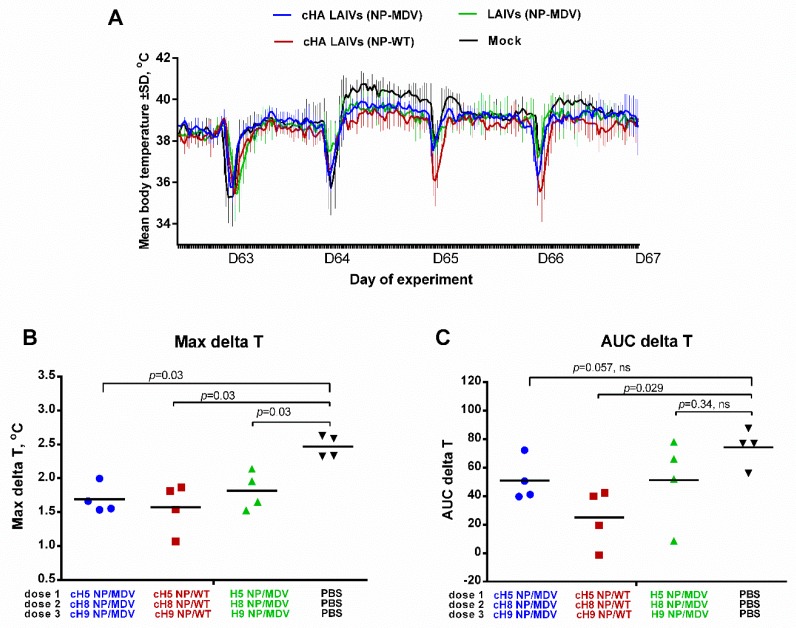
Body temperature of immunized ferrets after challenge. (**A**) Variation in body temperature over the challenge phase. The several sharp decreases in temperature are the result of sedation during challenge (D63) and collection of nasal wash fluids. (**B**) Maximal increase in body temperature over the challenge phase. (**C**) Area under the curve of delta T relative to baseline temperature for each ferret over the challenge phase. Statistical differences with mock-vaccinated group are indicated (Mann-Whitney U test); ns = not significant.

The body temperature of the ferrets in the challenge phase is shown in Figure 10A. There was a significant difference between all three vaccine groups and the control group in the maximal delta T parameter (Figure 10B), suggesting that the vaccinated animals were protected against severe fever. However, in terms of the area under the curve, the only significant difference was between the cHA LAIVs (NP-WT) group and the controls (Figure 10C). These data suggest that, while all vaccine regimens reduced fever similarly, the duration of fever was significantly shorter in animals that received LAIVs containing chimeric HA and WT NP.

#### 3.4.4. Histopathological Analysis of Upper and Lower Respiratory Tract

**Figure 11:**
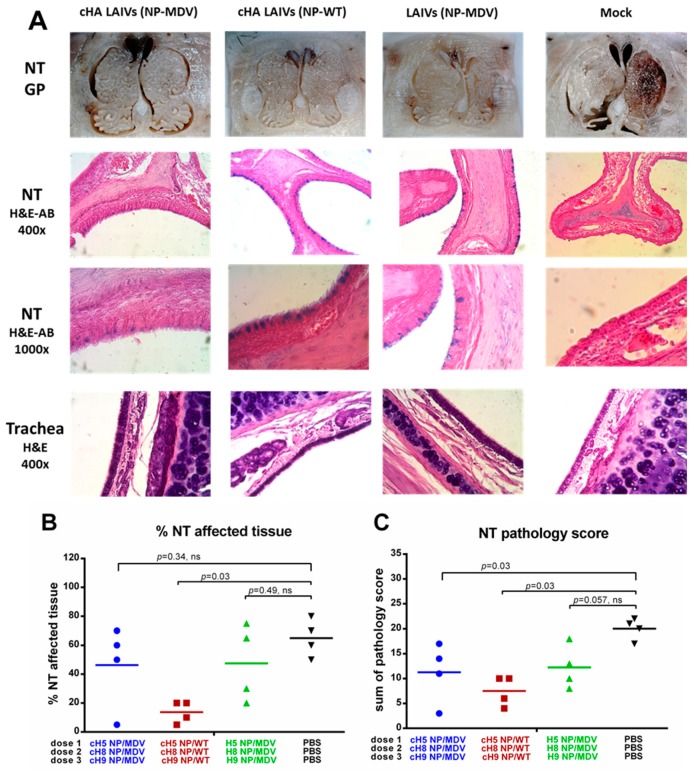
Pathology of the upper respiratory tract of immunized ferrets four days post-challenge. (**A**) Gross pathology (GP) of nasal turbinates (upper panel). Histopathology of NT (middle panel) and trachea (lower panel). (**B**) Percentage affected nasal turbinate tissues. (**C**) Semi-quantitative analysis of NT infection pathology. H&E—haematoxylin and eosin. AB—alcian blue. Statistical differences with mock-vaccinated group are indicated (Mann-Whitney U test); ns = not significant.

Analysis of pathological changes in URT tissues demonstrated that all vaccinated groups were protected from damage to respiratory epithelium after H1N1pdm09 challenge: mucus-producing goblet cells (stained with alcian blue stain) were preserved in NTs of LAIV-vaccinated ferrets, whereas these cells were absent in the mock-vaccinated group (Figure 11A). However, semi-quantitative analysis of pathological changes in nasal turbinates, and estimation of the percentage of affected tissue, revealed significant protection only in the two groups given LAIV that expressed chimeric HAs (Figure 11B,C). Interestingly, only the cHA LAIVs (NP-WT) group was significantly protected in terms of both the percentage of affected tissue and the total pathology score (Figure 11B,C). A semi-quantitative analysis of the infection pathology of individual NT specimens is given in Supplementaly Materials Table S1.

Histological examination of ferrets’ trachea showed few or no pathological changes. Minimal to moderate glandular, subepithelial and intraepithelial mononuclear infiltrates were defined. Rarely damage to cilia, epithelial hypertrophy and hyperplasia were found; however, there were no significant differences between the groups.

**Figure 12:**
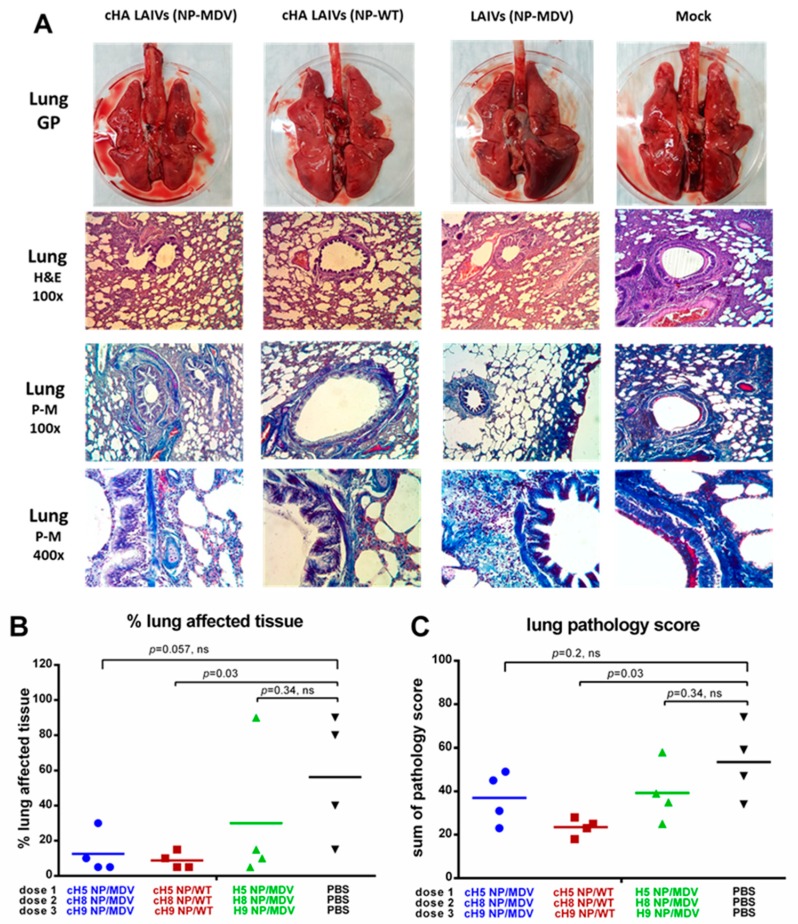
Pathology of the lower respiratory tract of immunized ferrets four days post-challenge. (**A**) Gross pathology (GP) of ferrets’ lungs (upper panel). Histopathology of lungs with different dyes (middle and lower panels). (**B**) Percentage affected lung tissues. (**C**) Semi-quantitative analysis of lung infection pathology. H&E—haematoxylin and eosin. P-M—picro-Mallory stain. Statistical differences with the mock-vaccinated group are indicated (Mann-Whitney U test); ns = not significant.

The lungs of the control ferrets showed severe pathological changes, characterized by moderate to strong bronchopneumonia and moderate interstitial and alveolar pneumonia (Figure 12A). There was regularly defined hyperemia of the alveolar septa with large hemorrhages, alveolar emphysema, necrosis and denudation of bronchial and bronchiolar epithelium. Alveolar and bronchial spaces were often filled with edema, debris, and large numbers of polymorphonuclear cells. Vasculitis and perivasculitis were defined.

The lungs of ferrets from the groups given cHA LAIVs (NP-MDV) and LAIVs (NP-MDV) had macroscopically visible emphysema foci and alveolar hemorrhages of varying severity. Exudate was observed in the lumen of the bronchi and bronchioles, in which lymphocytes and mononuclear cells predominated. Mild to moderate signs of catarrhal bronchitis, peribronchitis, bronchiolitis, perivasculitis and vasculitis were seen. Damage to pulmonary tissue was manifested as moderate lymphocytic and mononuclear infiltration in the interstitial tissue of the respiratory department, hyperemia of alveolar septa and alveolitis, shown as thickening of alveolar septa, and diffuse inflammatory infiltration. Focal hemorrhages and alveolar emphysema were defined (Figure 12A).

In the cHA LAIVs (NP-WT) group, morphological changes included minimal or slight thickening and hyperplasia of bronchial and bronchiolar epithelium, often without a microscopic picture of bronchitis and bronchiolitis. No exudate was detected in the lumen of the bronchi and bronchioles. In peribronchial areas, interstitial and alveolar tissues, there was predominantly minimal to moderate lymphocytic and mononuclear infiltration. Less frequently, there was necrotic damage to the bronchial epithelium, accompanied by moderate or severe lymphocytic infiltration (Figure 12A). Picro-Mallory staining of the lungs showed fibrin deposits in the lumen of the bronchi and the alveolar tissue. Detection of a large amount of fibrin and severe inflammatory infiltration of lung tissue in animals of the control group indicated the development of severe exudative fibrous bronchitis and alveolitis. In all vaccinated animals, fibrin was determined less frequently or was absent in the foci of inflammation (Figure 12A).

In conclusion, all groups of vaccinated animals showed fewer pathological changes and a lower percentage of affected tissue in the lungs than the control group. However, differences in the pathology score and percentage of affected tissue were statistically significant only for the cHA LAIVs (NP-WT) group, most likely because of the low number of animals analyzed (Figure 12B,C). A semi-quantitative analysis of the infection pathology of individual lung specimens is given in Supplementary Materials Table S2.

We conducted a correlation analysis of antibody immune responses in ferrets after immunization with the outcomes of heterologous influenza viral challenge. Some representative results are shown in Supplementary Materials. Homologous antibody responses (i.e., reacting with one of the LAIV viruses or HA protein with homologous head domain) correlated poorly with the increase in body temperature after heterologous viral challenge (Spearman r ~ −0.30, *p* > 0.15, Supplementary Materials Figure S1). In contrast, an endpoint ELISA with chimeric cH6/1 protein used as antigen revealed the population of antibody that correlated with the AUC delta T parameter (Spearman r = −0.74, *p* = 0.007). The best correlation was observed for cH11/1N1 microneutralization antibody titer and the AUC delta T (Spearman r = −0.82, *p* < 0.0001, Supplementary Materials Figure S1). Similar results were found for other pathological outcomes of influenza virus infection, such as lung histopathology score (Supplementary Materials Figure S2) and viral titer in lungs (Supplementary Materials Figure S3), suggesting that the stalk-reactive broadly neutralizing antibody played a significant role in protecting against a heterologous influenza virus challenge.

Overall, the universal LAIV candidates expressing chimeric HAs are efficient inducers of broadly reactive HA stalk-targeted antibody and efficiently protect ferrets against a high dose of heterologous influenza virus, thus demonstrating their potential for further clinical development.

## 4. Discussion

Because influenza A viruses are constantly evolving, vaccination with classical influenza vaccines is not always fully effective. Circulating strains are often antigenically significantly different from the seasonal vaccine components [49,50]. In addition, the causative agent of the next influenza pandemic cannot be predicted with complete certainty, meaning that the population may be unprotected against the new virus. As a result, the development of broadly protective influenza vaccines against different subtypes of influenza A virus has been a priority for some 20 years.

There have been various studies showing that influenza vaccines or other immunogens, such as recombinant proteins, that contain chimeric HA proteins (i.e., identical stalk domains and different globular head domains) are able to elicit significant HA stem-reactive antibody levels. As a result, such vaccines provide significant heterosubtypic protection, thus serving as a prototype universal influenza vaccine [13,51,52,53,54,55]. Recently, Nachbagauer and co-authors demonstrated in a ferret model that a cHA-based LAIV was a better priming vaccine for inducing high levels of cross-reactive HA stalk antibody after a boost with an inactivated influenza vaccine, regardless of the LAIV platform used [15,16]. We have previously demonstrated that sequential immunization with two or three doses of cHA-based LAIVs can induce HA stalk-reactive antibody that enhances heterosubtypic protection in mice, compared with heterologous prime-boost immunization with classical LAIVs [17]. In the present study, we assessed the safety, immunogenicity and cross-protective efficacy of such cHA-based LAIVs in a ferret model. We also tested modified LAIV in which the NP was replaced from the Len/17 MDV by the NP from the current H1N1pdm09 virus.

The rationale for including recent NP in the LAIV genome composition has been discussed in the context of our previous studies in a mouse model [37,38], and in in vitro study of human donor peripheral blood mononuclear cells [36]. In this preclinical ferret study, for the first time the two strategies for enhancing the cross-protective potential of traditional LAIVs were combined, and sequential immunizations with the vaccines expressing cHAs and WT NP were compared with classical LAIVs and with cHA-based LAIVs with classical NP.

In vitro characterization of the LAIV viruses showed that they all preserved the *ts* phenotype, which is important for the maintenance of attenuated phenotype of the vaccine viruses. However, the inclusion of chimeric HA and/or WT NP genes in some cases affected the *ca* phenotype and/or their ability to replicate efficiently in MDCK cells. Similar effects have been demonstrated previously for the engineered cHA-based LAIVs [17], as well as for the 5:3 reassortant viruses bearing WT NPs [38]; in all the studies these effects were strain-specific. Despite these variations in virological characteristics, all LAIV candidates used in the mouse studies were attenuated, i.e., did not grow in the lungs, but were able to replicate in the upper respiratory tract, inducing protective immunity. In the current ferret study, we could not assess LAIV virus replication in the lower respiratory tract because of the limited number of animals. However many previous studies of Len/17-based LAIV viruses have confirmed the attenuated phenotype of these viruses [47,56,57]. The vaccine viruses were able to replicate in the upper respiratory tract, inducing humoral immune responses, but no clinical signs of disease were registered after immunization with any of the LAIVs tested. These findings indicate that the three-dose immunization schedule is safe, regardless of the LAIV virus composition tested in the trial.

In contrast to our previous mouse study [17], the ferrets developed lower HAI titers to the homologous viruses used for immunization. In mice, a significant increase in anti-H5N1 HAI antibody was seen in all groups that received H5 HA-containing LAIVs as the first dose [17]. In the ferret study, anti-H5N2 HAI antibodies and anti-H5N2 serum IgG antibodies were much lower than in the mouse study. Most probably, in ferrets a single dose is not sufficient to induce high homologous antibody to avian influenza viruses [56], while the other LAIVs bearing heterologous HA head domains were unable to boost the HAI antibody. Nevertheless, the most important immunological result is that only cHA-containing LAIVs were able to induce HA stalk-reactive IgG antibody, which is in agreement with our previous findings in mice [17] and with reports of other authors [15,16]. In addition, we demonstrated that the antibodies induced by cHA-expressing LAIVs possess neutralizing activity, and that these broadly neutralizing antibody titers were well correlated with the protection of ferrets against pathology induced by a heterologous influenza virus challenge.

The main purpose of this preclinical trial was to demonstrate that the replacement of the HA and NP genes in classical LAIVs with chimeric HA and WT NP genes did not affect the attenuated phenotype of the vaccines and that the cHA-based LAIV candidates are advantageous in terms of inducing cross-protective efficacy compared with classical LAIVs. Our previous comparative ferret study of H3N2 LAIVs containing NP from MDV or wild-type virus did not detect significant differences in the cross-protective efficacy of the vaccines, although there was a slightly lower challenge virus titer in the URT in the WT NP LAIV group. This was probably because the challenge virus failed to replicate in lungs and did not cause significant pathology [58]. In contrast to that study, here we used a high-dose viral challenge that induced significant pathology and clinical manifestations of disease in the control animals, allowing the protective potential of the vaccines to be evaluated through a number of parameters.

Overall, all three vaccination regimens protected animals from clinical manifestations of disease: immunized ferrets did not lose weight and did not present clinical symptoms such as sneezing and change in behavior, and their maximal temperature increase over the challenge phase was significantly lower than in the control group. These data confirm previous findings that even classical LAIVs afford partial protection against heterologous/heterosubtypic influenza virus challenge [59,60]. Despite this protection against clinical symptoms, the immune responses induced by the sequential immunizations were not sterile, i.e., challenge virus was isolated from the upper and lower respiratory tract of challenged animals. Although challenge virus replication in the URT was significantly lower than in the mock-vaccinated group for all three vaccine groups, only the cHA-containing LAIVs were able to significantly reduce viral titer in the trachea. Strikingly, only the LAIVs containing both cHA and WT NP protected against pulmonary virus replication.

A recent study by Nachbagauer et al. [15,16] found that a cHA LAIV prime followed by a cHA IIV boost induced a broad antibody immune response that fully protected ferrets from replication of heterologous virus in lungs. In that study, in addition to HA-reactive antibody, the protection could have been afforded by anti-NA antibody, since the viruses contained the NA of H1N1pdm09 virus. In our study, all LAIV candidates contained NA from an irrelevant strain, Len/17 H2N2 virus, to avoid any impact of anti-NA antibody on protection. The inability of our vaccination regimens to fully protect ferrets against challenge virus pulmonary replication might be related to the lower serum IgG antibody titers and the higher dose of the challenge virus.

In line with the virological findings, other observations also suggest that simultaneous inclusion of chimeric HA and wild-type NP in LAIV viruses provides better protection against pathological changes after challenge with high-dose heterologous virus. The most straightforward and objective clinical observation is temperature increase over the challenge phase. This was registered with implanted temperature recorders, which measure body temperature every 30 min. The resulting data array allows precise assessment of temperature increase using the AUC delta T parameter. Strikingly, despite similar Max delta T values for all three vaccine groups, the AUC values differed significantly between groups, with the lowest value being observed for the cHA LAIVs (NP-WT). These data suggest that, while fever was similarly reduced in all test groups, the duration of fever was significantly shorter in animals that received LAIVs containing both cHA and WT NP gene; this may be associated with CTL immunity. These findings were further confirmed by histopathological analysis of upper and lower respiratory tract tissues of challenged ferrets: a significant reduction in pathology scores, relative to the control group, was detected only for the group given cHA-containing LAIVs, with maximal protection observed in the cHA LAIVs (NP-WT) vaccine group. Overall, the virological and pathology findings suggest the following hierarchy in the protective efficacy of the vaccine groups: cHA LAIVs (NP-WT) > cHA LAIVs (NP-MDV) > LAIVs (NP-MDV). It should be noted that the differences in virological and pathological outcomes between the three vaccine groups were not significant, which may be related to the small number of ferrets in each group and genetic diversity of the animals. Similar results were demonstrated in our previous study of cHA-expressing LAIVs in mice: for some heterologous challenge viruses no significant differences were observed for the lung viral titers between cHA and classical LAIV groups, whereas for other viruses (H1N1 and H6N1) significant differences were achieved, suggesting better cross-protective potential of the cHA-based LAIVs over the classical counterparts [17].

The primary limitation of this study is that T-cell immunity was not analyzed for the immunized ferrets. This was because the assays were not available at the time the study was performed. In recent years, great strides have been made in characterizing T-cell immune responses to influenza infection in ferrets, and some reagents and standardized procedures have been established [61,62,63]. A recent study by Liu et al. [64] measured the NP-specific T-cell immune responses in peripheral blood mononuclear cells of ferrets immunized with cHA-expressing LAIVs (the vaccines were almost identical to our cHA LAIV NP-MDV group with Len/17 strain used as an attenuated backbone) after stimulation with Cal09 NP-specific 15-mer peptides overlapped by 11 amino acid. Strikingly, there was no significant increase in the population of IFNγ-secreting CD4 and CD8 T cells for any of the vaccination regimens, whereas significant T-cell responses against HA stalk-based epitopes were seen. One possible reason for the lack of NP-specific T-cell responses is that they were measured against recent influenza virus pH1N1, whereas ferrets were immunized with the vaccines containing NP of influenza virus isolated in 1957. Although there is approximately 90% homology between the two virus NP amino acid sequences, some of the mutations may be located within the T-cell epitopes and significantly affect their immunogenicity, which is especially important in light of the observation that CTL epitopes of influenza virus NP are under selective pressure and many of the non-synonymous mutations occur in the CTL epitope regions of this protein [32,65].

Nevertheless, it is unlikely that the results of the study of T-cell immune responses in ferrets can be directly translated into humans and predict the fine differences in the NP epitope-specific T-cell responses, both because of the differences in T-cell immunology between humans and animals, and in light of the observed distinct patterns of reactivity between individual animals as a result of the heterogeneity at the MHC locus within commercial populations of ferrets [66]. Since the rationale for the replacement of the NP gene in the LAIV virus is that some human T-cell epitopes in the NP of Len/17 strain are no longer present in recent influenza viruses, only comparative clinical trials of LAIVs with different NP on HLA-typed individuals can prove the hypothesis that the replacement of NP is beneficial for the induction of T-cell responses more relevant to current infections.

A possible obstacle to the widespread use of this strategy in clinical practice is a long period of immunization (63 days). Traditionally, to ensure the induction of high antibody titers after the second immunization, 3–4 weeks intervals are used between the two vaccinations. Although all preclinical studies of cHA-based LAIVs used a 3-week interval [15,16,64], ferret studies of some pandemic LAIVs showed that antibody responses peaked on the 14th day after the first vaccination [47,57], so it is possible that the antibody against the HA-stem can be elicited using the 2-week interval regimen. In addition, even two doses of cHA-based LAIVs could induce HA stalk-specific antibody and enhance cross-protective potential of the vaccines, both in mice [17] and ferrets [64]. Another important question is how long can the protective level of these antibodies be maintained. Although the study design did not include the assessment of the durability of the induced cross-neutralizing antibody, studies in humans suggest that anti-HA stalk antibody, once elicited, may persist for years [67].

## 5. Conclusions

In summary, we conducted a preclinical study of universal influenza vaccine candidates generated on the basis of licensed LAIVs. Comprehensive analysis of safety, immunogenicity and protective efficacy of cHA-based prototype universal LAIVs in a ferret model provided evidence that such vaccines are highly promising for further clinical development. The results indicate that the two sets of cHA-expressing LAIVs (carrying NP from MDV or WT virus) merit further evaluation in a phase I clinical trial to assess their ability to induce cross-reactive stalk-based antibody and NP epitope-specific CD8 T-cell responses in humans.

## Figures and Tables

**Table 1:** Virological characteristics of LAIV viruses used in this study.

LAIV Virus	Virus Titer in Eggs, log_10_EID_50_/mL			Phenotype	Virus Titer in MDCK Cells, log_10_TCID_50_/mL
	33 °C	38 °C	26 °C		
H5 NP/MDV	9.2 ± 0.3	2.7 ± 0.5	6.5 ± 0.7	*ts/ca*	8.6 ± 0.3
H8 NP/MDV	7.6 ± 0.8	1.6 ± 0.2	5.5 ± 0.4	*ts/ca*	6.0 ± 0.8
H9 NP/MDV	8.4 ± 0.5	1.7 ± 0.6	5.5 ± 0.8	*ts/ca*	7.4 ± 0.5
cH5 NP/MDV	8.6 ± 0.3	2.2 ± 0.5	6.3 ± 0.1	*ts/ca*	7.3 ± 0.3 ^†^
cH8 NP/MDV	7.7 ± 0.7	1.2 ± 0.2	5.1 ± 0.9	*ts/ca*	5.8 ± 0.4 ^‡^
cH9 NP/MDV	8.9 ± 0.4	2.0 ± 0.6	6.5 ± 0.7	*ts/ca*	8.2 ± 0.0
cH5 NP/WT	7.5 ± 0.4 ^†^	1.5 ± 0.4	3.0 ± 0.3	*ts/non-ca*	6.4 ± 0.4 ^†^
cH8 NP/WT	8.1 ± 0.7	2.2 ± 0.8	5.2 ± 0.7	*ts/ca*	7.0 ± 0.2
cH9 NP/WT	7.8 ± 0.7	2.1 ± 0.4	5.2 ± 0.8	*ts/ca*	5.2 ± 0.3 ^†^

^†^*p* < 0.05 compared with the corresponding control LAIV virus with classical HA and NP from MDV. ^‡^ *p* < 0.05 compared with the cH8 NP/WT LAIV.

## Supplementary Materials

The following are available online at https://www.mdpi.com/2076-393X/7/3/61/s1, Figure S1: Correlation of some representative antibody immune responses of ferrets after immunization with body temperature increase measured by the area under the curve during the challenge phase, Figure S2: Correlation of some representative antibody immune responses of ferrets after immunization with lung pathology score defined during histopathological analysis of lung tissue sections, Figure S3: Correlation of some representative antibody immune responses of ferrets after immunization with viral titer in lung on day 4 post-challenge, Table S1: Individual nasal turbinates pathology scores for immunized ferrets after challenge with H1N1pdm09 virus, Table S2: Individual lung pathology scores for immunized ferrets after challenge with H1N1pdm09 virus.
