# Supplementary material for: Sequential Immunization with Universal Live Attenuated Influenza Vaccine Candidates Protects Ferrets against a High-Dose Heterologous Virus Challenge
Source: Vaccines (Basel). 2019 Jul 8;7(3):61. doi: 10.3390/vaccines7030061 (PMC6789596; doi:10.3390/vaccines7030061)
Supplement: Supplementary file 1 [file vaccines-07-00061-s001.pdf]

vaccines

MDPI

Article

Sequential Immunization with Universal Live Attenuated Influenza Vaccine Candidates Protects Ferrets against a High-Dose Heterologous Virus Challenge

Irina Isakova-Sivak, Victoria Matyushenko, Tatiana Kotomina, Irina Kiseleva, Elena Krutikova, Svetlana Donina, Andrey Rekstin, Natalia Larionova, Daria Mezhenskaya, Konstantin Sivak, Arman Muzhikyan, Anastasia Katelnikova and Larisa Rudenko

Supplementary Materials

Table S1. Individual nasal turbinates pathology scores for immunized ferrets after challenge with H1N1pdm09 virus.

Vaccine group	Animal ID	Affected tissue (%)	NT pathology parameters					Total NT pathology score
			Epithelium damage	Hyperemia	Lymphocytic infiltrate	Exudate	Edema	
cHA LAIVs (NP-MDV)	1.1	70	3	1	3	4	3	14
	1.2	5	0	2	1	0	0	3
	1.3	50	2	2	2	3	2	11
	1.4	60	3	3	3	4	4	17
cHA LAIVs (NP-WT)	2.1	5	1	1	1	1	0	4
	2.2	20	2	3	2	1	2	10
	2.3	20	2	2	3	2	1	10
	2.4	10	1	1	2	1	1	6
LAIVs (NP-MDV)	3.1	65	3	4	3	4	4	18
	3.2	20	1	3	1	1	2	8
	3.3	30	2	3	1	2	2	10
	3.4	75	2	3	3	1	4	13
Mock (PBS)	4.1	70	4	3	4	5	5	21
	4.2	80	4	5	4	5	4	22
	4.3	60	4	3	3	4	3	17
	4.4	50	4	4	4	4	4	20

Table S2. Individual lung pathology scores for immunized ferrets after challenge with H1N1pdm09 virus.

**Vaccine group**	**Animal ID**	**Damage (0-5)**												**Inflamination (0-5)**								**Total lung pathology score**
		Epithelium hypertrophy		Epithelium hyperplasia		Pseudo squamous epithelium		Epithelium necrosis		Hypertrophy pneumocyte type2	Hyperemia septa	Alveolar emphysema	Alveolar hemorrhages	Bronchitis	Bronchiolitis	Peribronchitis	Peribronchiolitis	Interstitial infiltrate	Alveolitis	Vasculitis	Perivasculitis	
		bronchi	bronchioli	bronchi	bronchioli	bronchi	bronchioli	bronchi	bronchioli													
cHA LAIVs (NP-MDV)	1.1	3	2	2	3	0	1	2	3	3	4	2	1	3	2	4	3	3	3	2	3	49
	1.2	2	1	1	2	0	0	0	0	3	3	2	1	1	1	2	2	4	3	1	2	31
	1.3	0	1	1	2	0	0	0	1	2	2	3	0	0	2	1	2	3	2	0	1	23
	1.4	3	2	2	1	0	0	3	3	3	1	4	1	4	4	3	3	3	2	1	2	45
cHA LAIVs (NP-WT)	2.1	0	0	2	1	0	1	0	0	2	2	2	0	0	1	1	0	2	2	0	2	18
	2.2	1	0	2	1	0	0	0	0	2	2	1	2	1	1	2	1	2	2	1	2	23
	2.3	0	1	1	1	0	1	0	0	3	1	3	0	1	1	2	2	3	2	1	2	25
	2.4	1	1	1	0	0	0	0	2	3	2	0	0	1	3	2	2	3	3	2	2	28
LAIVs (NP-MDV)	3.1	0	2	0	1	0	0	0	0	3	2	2	0	1	1	1	2	4	3	1	2	25
	3.2	2	3	2	2	0	1	1	2	3	2	3	0	2	2	2	1	3	3	2	3	39
	3.3	1	2	1	3	0	2	0	1	3	2	1	3	2	1	2	2	3	3	1	2	35
	3.4	2	2	2	2	0	0	3	4	4	2	4	3	4	5	3	4	4	3	3	4	58
Mock (PBS)	4.1	3	3	2	3	0	2	2	4	4	4	3	1	2	4	3	4	5	4	3	3	59
	4.2	2	2	3	4	0	2	0	0	4	3	2	2	1	1	2	0	3	2	0	1	34
	4.3	2	2	3	3	1	1	0	2	3	3	3	4	2	2	2	2	4	3	2	3	47
	4.4	3	3	4	3	2	2	3	3	4	4	4	2	4	5	5	5	5	5	4	4	74

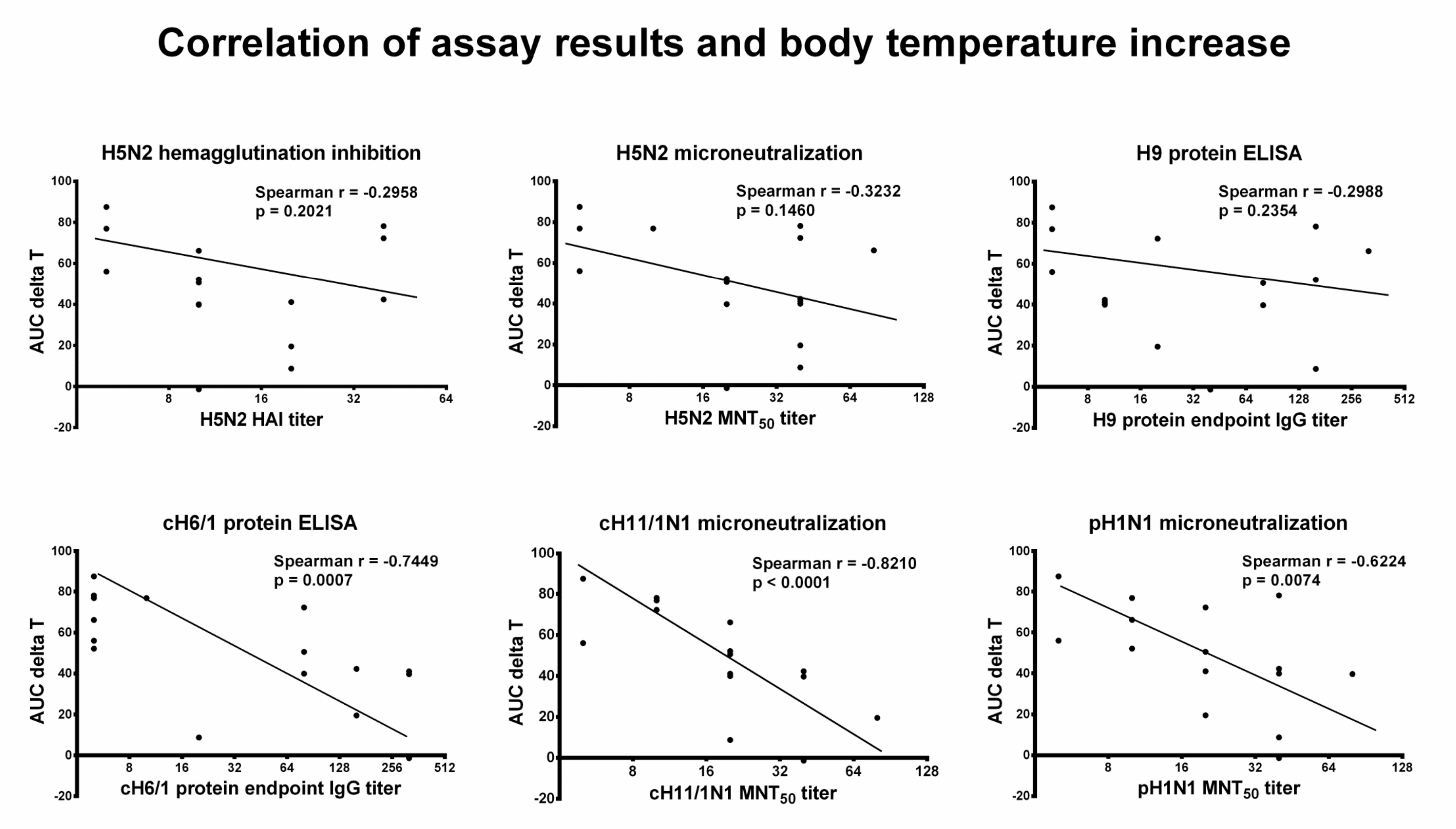

Figure S1. Correlation of some representative antibody immune responses of ferrets after immunization with body temperature increase measured by the area under the curve during the challenge phase.

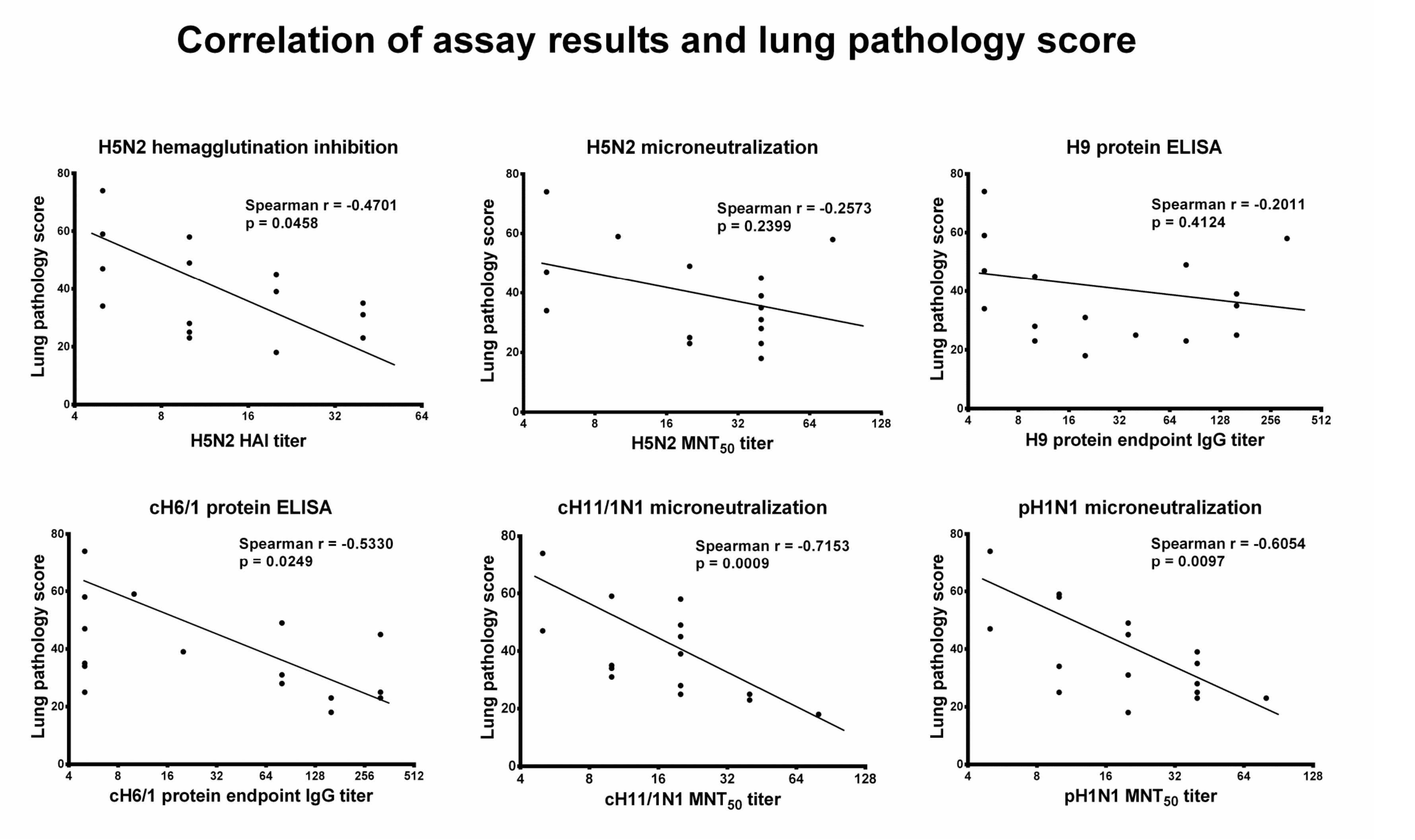

Figure S2. Correlation of some representative antibody immune responses of ferrets after immunization with lung pathology score defined during histopathological analysis of lung tissue sections.

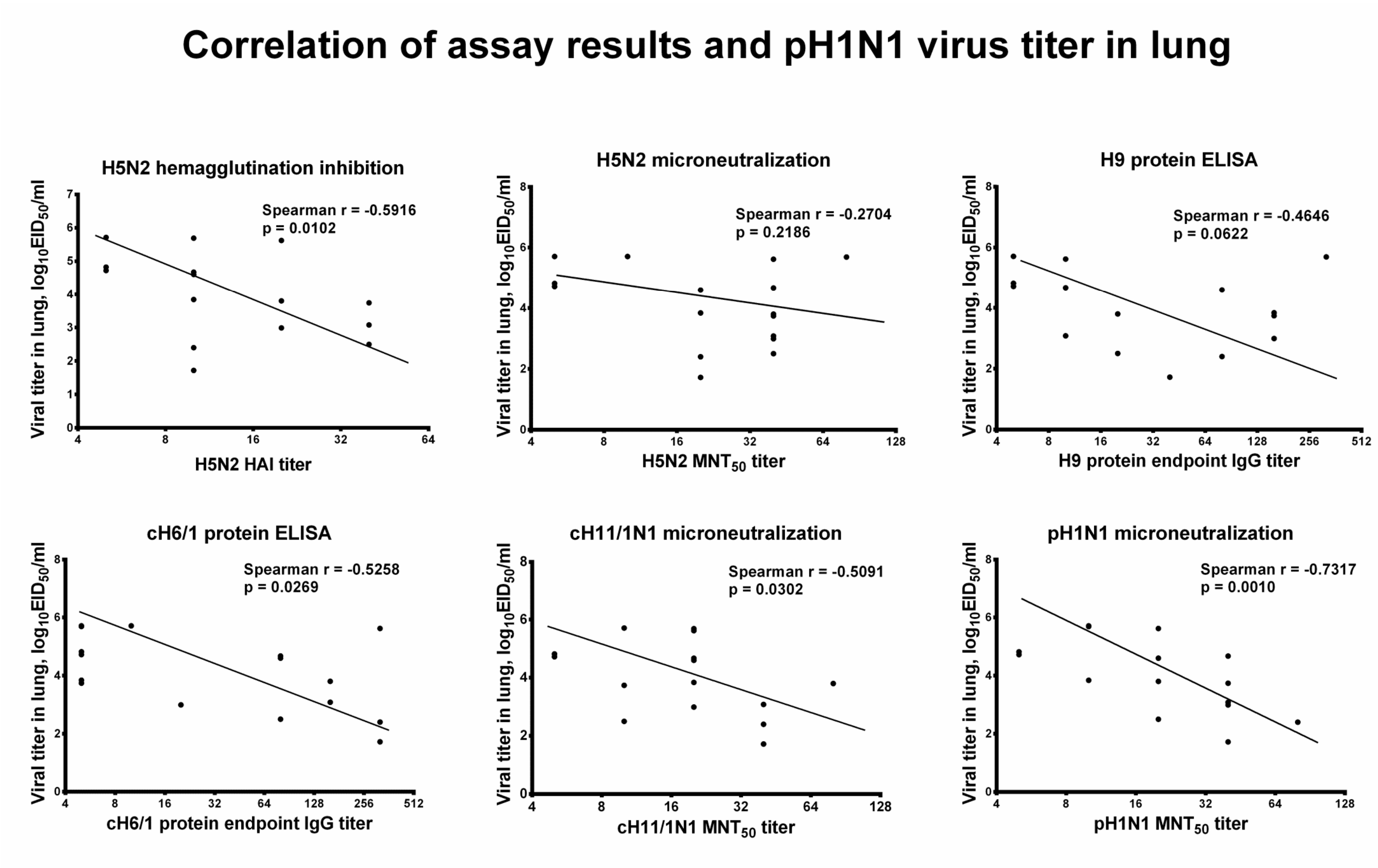

Figure S3. Correlation of some representative antibody immune responses of ferrets after immunization with viral titer in lung on day 4 post-challenge.

© 2019 by the authors. Submitted for possible open access publication under the terms and conditions of the Creative Commons Attribution (CC BY) license (http://creativecommons.org/licenses/by/4.0/).
